# Tableau (version 2020.3)

**DOI:** 10.5195/jmla.2021.1135

**Published:** 2021-01-01

**Authors:** Lynly Beard, Negeen Aghassibake

**Affiliations:** 1 lynly@uw.edu, Senior Assistant Librarian, Health Sciences Library, University of Washington Libraries, Seattle, WA; 2 negeena@uw.edu, Assistant Librarian, Health Sciences Library, University of Washington Libraries, Seattle, WA

Tableau is a data visualization tool that can be used to help libraries analyze data. It can also be an important tool for researchers who want to supplement their text with engaging visual elements to make their data and analysis easily understood, not only in their departments, but also by investors, grant funders, other academic audiences, and the general public. This is an update of the 2016 review of Tableau version 9.1 by Ariel Deardorff [1]. A new edition, Tableau 2020.3, has been released with new features to assist users in creating more sophisticated visualizations with greater ease.

Tableau runs on both Mac and Windows platforms. The minimum requirements to use the software will likely be met with most devices that also run software such as the Microsoft suite of tools. Tableau lists detailed technical specifications that are required to run the software on their website [2]. Although Tableau does not require any programming or technical training, practice is essential.

There are several different versions of Tableau to meet user needs. Tableau Public is free, though any saved data are stored on the Tableau Public server. Users should take care to not save any sensitive data using this version. Tableau Desktop (paid) allows users to save visualizations locally on their computers, and Tableau Server (paid) allows multiple users to save their work to their own servers. Tableau Online (paid) also offers a private server, though the server would be Tableau's. For users who want to share their work only with others such as investors, grant funders, administrators, faculty, or students, Tableau Reader is an option. Reader is free to install, and end users can interact with visualizations on their own devices without needing a license. Ultimately, the decision of which version of Tableau to use depends on the sensitivity of the data and collaboration needs.

The intended audience for Tableau is quite broad, although it is heavily marketed to businesses and somewhat less so to academia. However, there are still opportunities for library staff to learn about and support their academic users with Tableau. Data visualizations in the *New York Times* and other popular press can make an idea or a data set more engaging for readers. In the same way, Tableau can make organizational or research data come to life for users and the groups with which they work. Academics who want to share their research with a wider audience may find that using data visualizations makes their work more intriguing and accessible: they can focus readers' attention on their conclusions and tell a story with both their words and their visuals.

Some examples of how library users and academic researchers have used these visualizations include epidemiologic surveillance and contact tracing, health care utilization analysis, and clinical research data analysis [3]. This can be important in appealing to audiences who may be unfamiliar with academic channels and languages. Library staff who have data visualization skills can better support their users who are interested in sharing their research data with a wider audience.

Tableau can also be used to help users who conduct research visualize the impact of their work [4]. Though Tableau may not always be the primary resource used to generate these visualizations (for example, network visualization tools can help construct charts showing bibliometric data), it can play a role in this type of analysis. Library staff who provide support for research impact may benefit from incorporating visualization capacity into their work with users.

Tableau can serve as a tool to analyze information about library services and collections. Some examples of data that can be visualized using Tableau include survey results, interlibrary loan records, and electronic resource usage. In addition, many libraries have created visualizations for their own administrative and strategic goals. These visual data can be used to analyze gate counts, head counts in library spaces, collection usage, and public services usage [5].

To introduce users to Tableau, free training videos on their website cover visual analytics, dashboards, and stories [6]. There are also Tableau Community Forums, where Tableau users can share ideas and answer questions. Tableau experts will often help answer questions. In addition, there are some city-specific Tableau User Groups that connect local users, and there are classes on LinkedIn Learning (formerly, Lynda.com), including beginner and intermediate training videos that walk through learning Tableau step-by-step and provide sample data. Finally, many universities have their own user groups for community discussion and enrichment.

Tableau guides users through its drag-and-drop interface and its “Show Me” function, which provides chart options, such as Gantt charts, bar charts, and maps. There are several chart options, though library data tend to be best visualized through more “traditional” bar and line charts. However, the “Show Me” function makes it easy to explore less common chart types and quickly test different types of visualizations.

Tableau 2020.3 has several new features, only some of which are mentioned here. One key obstacle to using Tableau in an educational environment is web accessibility for users with disabilities. Since the 2016 version of Tableau, a few improvements have made Tableau more web accessible. For example, date filters in Tableau are now accessible by keyboard and screen readers, which can help facilitate exploration of the data for those who use visual assistive technology. Also, alternative text can now be added to images used in dashboards. While these improvements have helped the accessibility of visualizations created using Tableau, they rely on the end user to understand how they function and how to implement them.

Tableau's new “Ask Data” feature allows users to type a question they want to ask of their data using natural language and have that question answered through automatically created visualizations. This can help library staff who are new to data analysis and visualization learn more about their data interactively.

Another new feature is Tableau Relationships, which makes it easier to connect multiple data sources. This is particularly useful for library work where data can be stored in more than one system; for example, gate count data may be in a vendor system, reference and instruction statistics may be in a spreadsheet, and collections usage could be in yet another system. Though it is still useful to learn about how to join data sources, Tableau Relationships simplifies the process.

Tableau is not the only interactive data visualization tool available on the market. Other resources function similarly, though their interfaces differ. Microsoft Power BI is an interactive data visualization tool that performs many of the same tasks as Tableau and has a drag-and-drop interface. It may be an option for those at institutions that subscribe to it through the Microsoft suite of tools. In addition, though Microsoft Excel is not primarily geared toward visualization, users can create simple charts and dashboards as well as add some interactive filters and features. Visualization capabilities are also included in Google Sheets, much like Excel, that allow users to create simple visuals by clicking on Insert Chart. Although Tableau has risen in popularity in the past few years, there are alternatives available for information professionals, and the decision about which tool to choose will depend on ease of use, accessibility, and funding.

Tableau 2020.3 provides tools that both librarians and their patrons may want to use to incorporate data visualizations into their research. Librarians will find it useful for internal purposes, and it will be a valuable offering for patrons who want to share their research with varied stakeholders in a simple and powerful fashion.
